# Esthetical Properties of Single-Shade and Multishade Composites in Posterior Teeth

**DOI:** 10.1155/2023/7783321

**Published:** 2023-09-12

**Authors:** Graziela R. Batista, Alessandra B. Borges, Rayssa F. Zanatta, Cesar R. Pucci, Carlos R. G. Torres

**Affiliations:** ^1^Missouri School of Dentistry and Oral Health – MOSDOH, A.T Still University – ATSU, Kirksville, Missouri, USA; ^2^Department of Restorative Dentistry, Institute of Science and Technology, São José dos Campos, São Paulo State University - UNESP, São Paulo, Brazil; ^3^Department of Dentistry, School of Health Sciences, University of Brasilia - UnB, Brasilia, Brazil

## Abstract

This study aimed to compare the aesthetic properties of posterior composite restorations made with a multishade (MS) or single-shade (SS) material. For that, 23 extracted human molars were used. The shade determination was performed, and the occlusal anatomy was registered by a custom-made stamp. Then, class I preparations were made, and each tooth was restored twice, using two different composites of MS/opacity layering material (Admira Fusion—Voco) and an SS/opacity bulk-fill composite (Admira Fusion X-tra—Voco). After finishing the first restoration with the MS material, a standardized picture was taken, and the restoration was removed. Then, the preparation was restored again with the other composite, obtaining a new picture. The pictures were randomly analyzed by 10 calibrated evaluators regarding color match using the FDI criteria. The evaluators were blinded to the restorative material used. Data were analyzed using an unpaired *t*-test and Wilcoxon test. The significance level adopted in the study was 5%. As a result, there were nonsignificant differences between the composites evaluated for color match, as well nonsignificant differences were obtained related to which restorative technique produced the best aesthetic results. Only for darker shades (A4, B4, and C4), most of the evaluators considered the restorations made with the MS material more aesthetic than with the SS, while for the other shades, nonsignificant differences were observed. Therefore, the choice of the SS and the MS/opacity composites does not influence the aesthetic outcome of posterior restorations for the lighter shades. However, the use of bulk fill material for dark shades is less favorable than the MS layering ones.

## 1. Introduction

Composite resins are considered nowadays the best option for direct restorations when an esthetic outcome is needed [1]. As the human tooth is a polychromatic structure and its color is a result of the light interaction with enamel, dentin, and pulp [2], these materials present differences in their composition to mimic some tooth optical properties, such as color, translucency, fluorescence, opalescence, and gloss [3, 4].

Tooth shade is mainly determined by the dentin, which is yellowish and opaquer than enamel. This latter is more translucent, contributing to observed tooth shade by the scattering of light wavelengths in the blue range [2, 5, 6]. The color of different areas of the tooth structures varies according to the enamel/dentin thickness ratio, and in order to reproduce the natural aspect of the tooth, manufacturers create restorative materials with different opacity levels. The optical congruence between the material and tooth tissues is accomplished by color layering, and the final color of a restoration is the result of the blend of several individual layers [7, 8]; so, on anterior teeth, the use of a single opacity composite can produce a color mismatch between the restoration and the remaining tooth structure. Thus, the so-called “chameleon effect,” or blending effect, is sought by clinicians, using multishade (MS)/opacities materials through layering technique, trying to produce a more esthetical restoration [9]. The blending effect refers to the smallest color difference observed between composites and dental tissues [10, 11].

Although the use of multiple opacity materials is mandatory in anterior teeth, the use of those at posterior teeth is sometimes considered unnecessary. Therefore, to simplify the restorative technique, some manufacturers proposed a universal shade material claiming to have a blending effect with the teeth [12–15]. The main advantage of these materials is an enhanced color adjustment potential (CAP), which can match different teeth colors [13]. CAP is a term that describes and quantifies the interaction between physical and perceptual components of blending [16]. Although the application of these materials in anterior teeth seems limited [12], recent evidence showed a good blending effect when using composites with greater CAP in posterior teeth, simplifying the shade matching and reducing the risk for color mismatch [16]. This proposal gained a higher impact with the development of bulk-fill composites, claiming to simplify the restorative technique using a small number or even a single increment [17]. The color-matching found in universal shade bulk-fill composites is attributed to their high translucency, reflecting the shade of the surrounding dentin walls [7, 18]. This behavior was also maintained when they were placed over nontranslucent materials used to cover a particular darker dentin [7].

Scientific evidence in the literature regarding the impact of MS or single-shade (SS) materials on posterior restorations related to the color match and esthetic results, mainly assessed by visual methods, is scarce. Therefore, the aim of this study was to compare the esthetical outcome, using the color match criteria, when a posterior restoration is made using an MS or an SS material. The null hypothesis is that this choice does not produce significant differences in the final restoration for any tooth shade selected.

## 2. Materials and Methods

### 2.1. Specimen Preparation

Twenty-three intact upper and lower human molars extracted for different reasons were collected and used in this study after approval from the local ethics committee. The total number of teeth selected following a previous pilot study. The teeth were cleaned with pumice and water and then immersed in ultrapure water at 5°C until required.

The tooth shade determination was performed by a single calibrated operator using the shade guide provided by the manufacturer of the nanohybrid composite used in this study (Admira Fusion Shade Guide, Voco), which follows the Vita classical shade guide (Vita Zanhfabrik, Bad Säckingen, Germany). The shade determination was performed inside a color-viewing light booth (Konica Minolta, Osaka, Japan) with a D65 light source. The shade guide was placed close to the occlusal surface, and the operator decided on the shade option closest to the natural tooth structure. This shade determination was used to select the enamel shade composite.

After that, the occlusal anatomy of each tooth was copied by a custom-made stamp using a transparent polyvinyl siloxane material (Registrado Clear—Voco, Cuxhaven, Germany). The use of this occlusal matrix allowed the final restorations to have the same shape and anatomy and minimized the excesses of resin-based composite in the final restorations.

Standardized class I preparations were made using a tapered-shaped diamond point with a flat end and rounded corners (No. 3131, KG Sorensen, Cotia, Brazil). The buccolingual width of the preparation was standardized in 4 mm between the cusp tips, with a depth of 4 mm from the pupal wall to the cavosurface angle. The pupal wall was flattened, and the external walls were performed divergent toward the occlusal surface, with all internal angles rounded. The final depth of the preparations was checked using a periodontal probe, and the with and length dimensions were checked with a digital calypter. A standard preparation device was used to guarantee the same dimensions at all cavities (APC 100, Odeme, Brazil).

### 2.2. Restorative Procedures and Standardized Pictures

The universal adhesive system (Futurabond U, Voco) was applied for all restorations and used in the self-etching mode, according to the manufacturer's instructions. Each tooth was restored twice, using either an MS nanohybrid composite (Admira Fusion, Voco) or a bulk-fill nanohybrid SS composite (Admira Fusion X-tra, Voco). Both materials presented the same filler content (84% w/w) and monomers composition (Ormocer based) but different opacities characteristics.  Table 1 presents the composition and characteristics of each composite tested.

**Figure 1:**
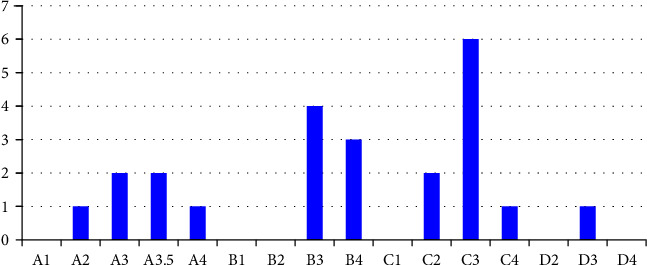
Distribution of the 23 teeth according to the Vita classical shade guide. Most teeth tested were classified as B3, B4, and C3.

The first restoration was made with the MS composite, which has two levels of opacity (dentin and enamel shades) and is indicated for layering technique with increments of 2 mm thickness. Therefore, this material was applied in two layers: the first one (2 mm) was intended to restore the lost dentin and was performed with an opaque shade (OA1, OA2, OA3, OA3.5). It was light cured for 20 s using an LED light curing unit (Valo Cordless, Ultradent Products, South Jordan, USA) with an emittance of 1,400 mW/cm^2^. The second layer (2 mm), intended to restore the enamel and recover the occlusal anatomy, was performed with the enamel shade material (A1, A2, A3, A3.5, A4, B1, B2, B3, C2, or D3). In this study, 1 tooth was A2, 2 were A3, 2 were A3.5, 1 was A4, 4 was B3, 3 were B4, 2 was C2, 6 were C3, 1 was C4, and 1 was D3 (Figure 1). When the tooth shade was A2 and C2, the OA2 dentin shade was used. When the shade was A3, B3, C3, and D3, the dentin shade used was OA3. When the shade selected was A3.5, A4, and C4, the dentin shade used was OA3.5. This color definition was decided through a pilot study.

**Figure 2:**
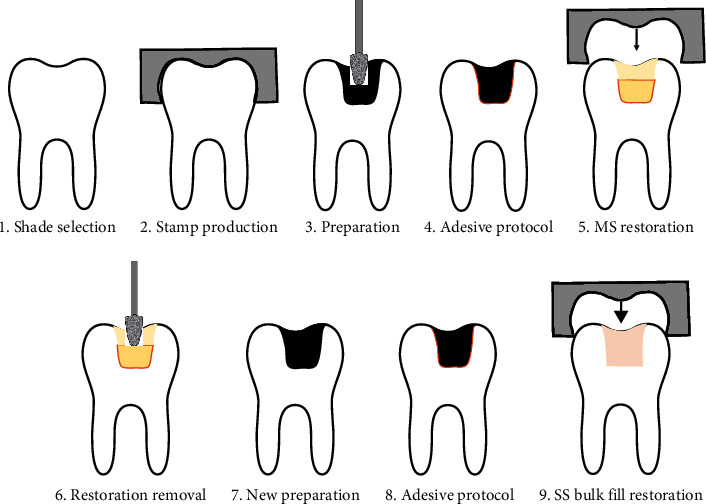
Schematic drawing of the restorative steps.

After placing the final composite layer, the clear occlusal stamp was pressed over the uncured composite to copy its anatomy, and light-curing was performed through it. Finally, the stamp was removed, and an additional light curing was performed for another 20 s.  Figure 2 shows a schematic drawing of these steps. The restorations were polished with an abrasive silicon carbide brush (Astrobrush, Ivoclar Vivadent, Lichtenstein), and the teeth were immersed in purified water for 7 days, allowing the postcuring of the material and the hydration of the tooth structure.

To standardize the surrounding color, a rubber dam was assembled in a frame, and a single hole was performed. Each single tooth was removed from the water and quickly placed into the hole, avoiding any dehydration that could influence color selection. The tooth was placed inside a color-viewing light booth (Konica Minolta, Osaka, Japan) with a light source simulating daylight. Standardized pictures of the occlusal surface were obtained using a digital camera (Rebel D, Canon Inc, Japan) coupled with a 100 mm macro lens (Canon) and a ring flash (Canon). The camera parameters settled were f/32, shutter speed 1/100, and ISO 400, and the flash was used in ETTL mode. The autofocus function was used to guarantee the ideal focus of the image. The camera was placed in a holder to have the long axis of the lens exactly 90° in relation to the occlusal surface. A gray reference card (WhiBal G7 Picture Flow LLC, USA) was used to perform white balance. The pictures were saved in TIFF format and then transferred to a computer, uncompressed and in high-quality.

After the first picture was taken, each tooth had its composite restoration removed with the same diamond bur used in the initial preparation. Special care was given to completely remove the composite without changing the shape and the depth of the cavity. Then, the same adhesive protocol used previously was applied, and the SS bulk-fill restorative material was placed (Figure 1), which has a universal shade and a single level of opacity. It is indicated for application in a single increment up to 4 mm thick. Then, the occlusal matrix stamp was placed over the composite with pressure, light cured for 20 s, and additionally cured after its removal, as previously described. The restorations were polished, and the teeth were immersed in ultrapure water for 7 days. After that, a second picture was obtained using the same parameters described for the first one.

### 2.3. Pictures Evaluation

Ten dentists with experience in operative dentistry were recruited and agreed to act as evaluators. They were previously calibrated to perform the esthetic properties evaluation for composite restoration according to the FDI criteria proposed by Hickel et al. [19, 20] (Table 2). Different scores were assigned to the evaluation of surface luster, surface and marginal staining, color match, and translucency. The evaluators were blinded about which restorative material was applied.

A total of 46 restorations were performed, and pictures were obtained, being two for each tooth, according to the restorative technique. The first step of the evaluation consisted of codifying each picture and transforming them into a presentation with one picture per slide (PowerPoint, Microsoft Office, Redmond, Washington, EUA). The images were individually shown to the evaluators, who attributed scores to them without knowing which material or which restorative technique was applied. All the evaluators observed the pictures on one of two screen-calibrated monitors. The number of times each restoration received each score by all evaluators was recorded.

In the second step of the evaluation, the two restorations of the same tooth were inserted side by side in the same slide, randomly assigned to the right or left side, and the evaluators were asked to indicate which restoration was considered more esthetic.

### 2.4. Illustrative Composite Translucency and Shade Evaluation

For illustrative purposes and to better understand and explain the results of the picture analysis, the translucency of the dentin and enamel shade (A2 and OA2) of the layering material, as well as the universal shade of the bulk fill material, were analyzed. Disk-shaped specimens of each material were prepared with a silicone mold with 6 mm diameter and 1 mm height. The material was light-cured and immersed in water for 24 hr. The surface and bottom of the samples were polished with P2400 and P4000 abrasive paper in an automatic polishing machine. After that, it was placed over a black background (Leneta Company WB, Mahwah, NJ, USA), and the *L*^*∗*^, *a*^*∗*^, and *b*^*∗*^ coordinates were obtained using the colorimetric spectrophotometer Vita Easyshade (Vita Zahnfabrik, Bad Säckingen, Germany). The shade determination was repeated over a white background. The translucency parameter (TP) was calculated as the color difference between the specimen over the black and white backgrounds [21]. The universal shade of the bulk fill composite, according to the Vita Classical shade guide, was also determined using the Vita Easyshade spectrophotometer.

### 2.5. Statistical Analysis

For each material (MS or SS), 23 restorations were analyzed by the 10 evaluators, which gave a different score for each esthetic property. The number of times each score was given to a restoration was compared between the materials using *t*-test for independent samples. The comparison between the two materials in relation to which produced a more esthetic restoration was analyzed by the Wilcoxon test separately for each evaluator. The significance level adopted in the study was 5%.

## 3. Results

The mean score and results of *t*-test for each esthetic property evaluated are shown in Table 3. Nonsignificant differences between the composites were observed for all scores.  Table 4 shows the results for the 10 evaluators about which composite produced the best esthetic result. The Wilcoxon test showed nonsignificant differences between the two composites for all evaluators.

**Figure 3:**
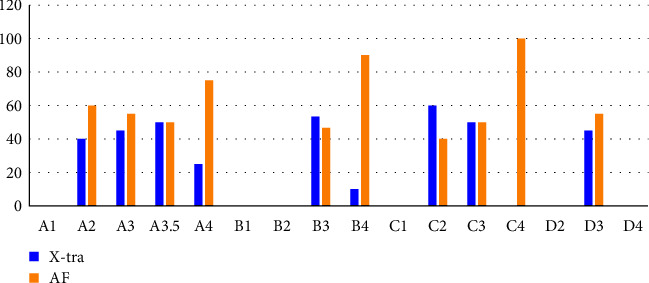
Percentage of restorations considered more esthetic for each composite, according to the Vita shades (X-tra = Admira Fusion X-tra —single-shade composite, AF = Admira Fusion—multishade composite). For the teeth considered A4, B4, and C4, the restoration with the multishade composite was considered more esthetical at most of the specimen's tested, evidencing that the single shade composite was less able to match the substrate.

**Figure 4:**
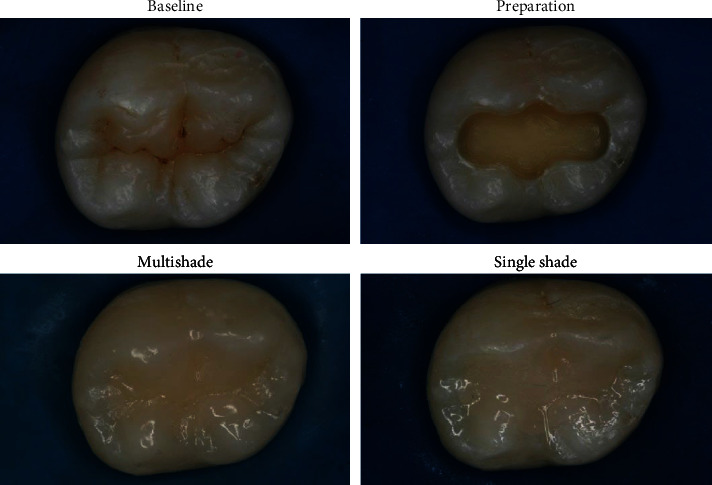
Tooth restored with the single shade and the multishade composites.

Figure 3 shows the shade distribution for all 23 teeth used in this study, according to the Vita Classical shade guide, and Figure 3 shows the percentage of the restorations with each material that was chosen as the more esthetic, considering the color of the tooth structure according to the Vita shade guide. It was observed that just for darker shades (A4, B4, and C4), most of the evaluators considered the restorations made with the MS material more esthetic than with the SS, while for the other shades, nonsignificant differences were observed. In addition, when all shades were analyzed together, nonsignificant differences between the materials were observed.  Figure 4 shows an example of a tooth restored with the SS and the MS composites. In relation to the TP, the dentin shade from the MS composite showed a value of 8.12 (0.022), while for the enamel shade, it was 9.27 (0.016). For the bulk fill SS material, the TP value was 11.82 (0.125). According to the Vita shade guide, the color of the SS composite tested corresponds to D2.

## 4. Discussion

In order to perform a good esthetic tooth restoration, the concepts of color and translucency must be understood by the clinician. Color is a psychophysical reaction to the light reflected by the surface of an object, which interacts with the visual system of the observer and is interpreted by the human brain [22, 23]. In the retina, three different light receptor cells, called cones, can absorb the reflected light in the wavelengths corresponding to blue, red, and green [22, 24]. Depending on the object's characteristics, the reflected light will vary, and the stimulus to the retina will result in different color sensations, creating a particular hue. The hue strength is named chroma. The retina also presents cells called rods, which are responsible for defining the value or the lightness of a color, ranging from black to white.

Usually, dentin presents a high color value, with typically less translucency, while enamel presents a much lower color value and more translucency [25]. A translucent object allows some light to be transmitted through it, while the ones that almost completely reflect or absorb the light are called opaque [25]. The combination of these three dimensions (hue, chroma, and value) gives the shade or tone of a color [26]. The characteristics of blocking or transmitting part of the light will change the amount of energy reflected to the observer, therefore affecting the final aspect of the object to the observer [24, 25]. The dental enamel is translucent and has low chrome and low value; therefore, most of the incident light will be transmitted and reach the dentin, which is opaquer, has a higher chrome and value, reflecting mainly the wavelengths corresponding to the yellow color back to the tooth surface, thus, resulting in the tooth color [2, 27]. In dentistry, the tooth color is generally referred to as shade. The tooth structure has different colors when thinking about chromaticity but also varies in relation to translucency depending on the area observed [25]. The thinner the enamel, the higher will be the chroma of the areas due to the higher influence of the dentin in the total shade aspect [25].

In this study, the null hypotheses tested were accepted as there were no significant differences regarding the esthetic parameters analyzed between the MS and SS composites tested for posterior restorations. To reproduce the tooth color, generally the dentist chooses the tooth shade related to its chroma and then selects composites with different levels of translucency to restore the lost enamel and dentin [25]. Otherwise, the use of just a highly translucent material to restore large preparations will reduce the light reflection and may give this area a grayish aspect, even when the correct chrome was selected [25]. This is commonly observed on class IV restorations without a remnant palatal wall, in which the light is lost in the inner part of the mouth [26]. However, in class I and II preparations, there is always the pulpal wall that will reflect some light. Therefore, in some situations, the use of an opaque composite may not be so relevant, and the use of a translucent composite, such as the bulk-fill ones, can produce a good esthetical outcome, which may partially explain the results observed in this study.

Color plays an important role in patient acceptance of a restoration; thus, the technology of smart chromatic materials, so-called universal, might be a scientific breakthrough in the field of dental materials, simplifying the shade selection and reproduction [16]. These materials are based mainly on structural color phenomena, which are the result of the fundamental optical processes of interference, scattering, or diffraction, and are claimed to be more accurate and stable than the pigment-based composites [12, 14]. Clinically, it is observed that the ability of these materials to take the color appearance of surrounding dental tissues after placement, thus improving esthetics, is material dependent [7, 10, 12–15]. The evaluation on the blending effect of universal shade bulk-fill composites to the human tooth have been evaluated [7, 13] and attributed to the high translucency of the material, reflecting the shade of the surrounding dentin and enamel walls, even when using different shades and translucency. Previously, Paravina et al. [10] showed that the blending effect was increased by increasing the translucency. These results corroborate with the findings from this study, as the universal shade composite tested showed satisfactory esthetic properties without differences from the MS one in terms of human perception. The TP analysis showed that the bulk universal shade is 45.56% more translucent than the dentin shade and 27.5% more translucent than the enamel shade of the layering material. The enamel shade is 14.16% more translucent than the dentin shade. There were no significant differences between the groups regarding the number of times each score was applied to restorations (Table 3) for all esthetic properties analyzed. This agrees with the results presented in Table 4, which shows that the blind evaluation by the observers did not show that the MS restorative procedure is different from the SS.  Figure 4 shows a tooth restored with the two techniques, resulting in both cases an adequate esthetic appearance.

In relation to the surface luster, all restorations were considered clinically acceptable (Table 4). This might be explained by the fact both composites present a similar composition. Both materials used in the present study are nanohybrid, with 84% inorganic filler particles, which were mainly barium aluminum borosilicate glass and silica (Table 1). The organic matrix of the composites tested were Ormocer molecules, an acronym for organic modified ceramic, which refers to a matrix of long backbone of inorganic silica with lateral organic chains, able to react during curing using conventional photoinitiators [28, 29]. These copolymers combine the benefits of organic polymers, such as flexibility and impact resistance, and of inorganic components, such as mechanical strength and chemical resistance [28]. Its large size reduces polymerization shrinkage and shrinkage stresses [29–32]. Still, this silicate nanoparticle technology enhances the chameleon effect, as it is able to blend and adapt to the surrounding tooth structure as they are smaller than the wavelengths of visible light, so neither diffracts nor refracts light, but allows it to pass through the material and bounce off the surrounding tooth structure color [14]. As the composition of both composites is similar (Table 1), the results of the polishing procedure were similar, and no differences were observed. In relation to the esthetic property color match, the manufacturer claims that the universal bulk fill composite presents a chameleon effect, being able to mimic color from the surrounding structure and underlying dentin. However, the percentage of restorations considered more esthetic for the darker teeth (A4, B4, and C4) was higher for the MS material, in which an adequate shade was available. According to the evaluation performed in this study, the Vita shade of the bulk fill material is similar to D2 and, therefore, closer to the lighter shades and would never match a darker remaining tooth structure around the preparation. In the case of darker shades, the color difference is too large, and the expected chameleon effect is not able to compensate for the difference. However, when all shades are analyzed together, non-significant differences are observed (Table 4). Regarding darkened shades, another expected problem would be for teeth with dark and sclerotic dentin on the pulpal walls or stained dentin in case of replacement of old amalgam restoration. In these cases, a thin layer of an opaque material, such as a flowable opaque composite, or a dentin replacement material, such as Biodentine (Septodont, Brazil), is advisable before the application. Recently, Miletic et al. [7] evaluated the potential of bulk fill and universal composites to match the tooth color when placed inside a posterior class I preparations with a bottom filled with dentin replacement materials and found that both composites performed similarly in terms of color difference, even after staining protocol applied. Regarding staining evaluation, the results of the present study shall be carefully analyzed as the restorations were not exposed to any staining solution, although a few examiners described some degree of color alteration within this item. That can be the result of a not perfect color match that mislead the examiners, as they just analyzed the pictures and were blinded about the procedures performed.

Nevertheless, despite the favorable results in terms of esthetics between both composites tested, the results from this study should be carefully analyzed as the esthetic evaluation was conducted without aging and with a small sample size within each color shade was tested. The composite discoloration is usually reported due to the presence of biofilm and extrinsic stain, surface or subsurface color variations due to the degradation of the resin matrix, or absorption of colorants within the material [33, 34]. Color change and discoloration is frequent a reason for a restoration replacement, being an important parameter to determine the longevity of a restoration, even in the posterior region [35]. Therefore, further studies should consider evaluating the effect of aging in these materials and its influence on the esthetics properties analyzed.

## 5. Conclusions

The choice for SS and the MS/opacity composites does not influence the esthetic outcome of posterior restorations for the lighter shades. However, for dark shades, the use of bulk fill material is less favorable than the MS layering ones.

## Figures and Tables

**Table 1:** Composition of the materials used.

Material	Indication	Content	Depth of cure	Filler content (% w/w)
MS: Admira Fusion	Multishade layering technique	Ormocer, photoiniciators, pigments, barium aluminum borosilicate glass, pyrogenic sílica (20–50 nm)	2 mm	84%

SS: Admira Fusion X-tra	Single-shade bulk fill technique	Ormocer, photoiniciators, pigments, barium aluminum borosilicate glass, pyrogenic sílica (20–50 nm)	4 mm	84%

Futurabond U	Universal adhesive	HEMA, Bis-GMA, HEDMA, MDP, UDMA, initiator, catalyst, and ethanol		/

**Table 2:** Esthetic properties according to the FDI criteria and described in Hickel et al. [19].

Scores	1. Surface lustre	2. Staining a. Surface b. Margin	3. Color match and translucency
1. Clinically excellent/very good	1.1 Luster comparable to enamel	2a.1 No surface staining 2b.1 No marginal staining	3.1 Good color match, no difference in shade and/or translucency

2. Clinically good (after polishing probably very good)	1.2.1 Slightly dull, not noticeable from speaking distance 1.2.2 Some isolated pores	2a.2 Minor surface staining, easily removable by polishing 2b.2 Minor marginal staining, easily removable by polishing	3.2 Minor deviations in shade and/or translucency

3. Clinically sufficient/satisfactory (minor shortcomings, no unacceptable effects, but not adjustable w/o damage to the tooth)	1.3.1 Dull surface but acceptable if covered with film or saliva 1.3.2 Multiple pores on more than one-third on the surface	2a.3 Moderate surface staining, not esthetically unacceptable 2b.3 Moderate marginal staining, not esthetically unacceptable	3.3 Distinct deviation but acceptable. Does not affect esthetics: 3.3.1 More opaque 3.3.2 More translucent 3.3.3 Darker 3.3.4 Brighter

4. Clinically unsatisfactory (but reparable)	1.4.1 Rough surface, cannot be masked by saliva film, simple polishing is not sufficient. Further intervention necessary 1.4.2 Voids	2a.4 Unacceptable surface staining; major intervention necessary for improvement 2b.4 Pronounced marginal staining; major intervention necessary for improvement	3.4 Localized clinical deviation that can be corrected by repair 3.4.1 Too opaque 3.4.2 Too translucent 3.4.3 Too dark 3.4.4 Too bright

5. Clinically poor (replacement necessary)	1.5 Very rough, unacceptable plaque retentive surface	2a.5 Severe surface and/or subsurface staining, generalized or localized, not accessible for intervention 2b.5 Deep marginal staining not accessible for intervention	3.5 Unacceptable. Replacement necessary

**Table 3:** Mean (SD) of the number of times mean each score was given to the 23 restorations with each material and the results of *t*-test.

Esthetic property	Score ^*∗*^	Mean		*t*-Test SS × MS (*p*-value)
		Single shade (SS)	Multi shade (MS)	
1. Surface luster	1.1	17.6 (2.4)	18.1 (3.8)	0.7301
	1.2.1	4.6 (2.1)	4.5 (3.5)	0.9393
	1.2.2	0.4 (0.5)	0.2 (0.4)	0.3553
	1.3.1	0.4 (0.5)	0.2 (0.4)	0.3553

2. Staining				
a. Surface	2.a.1	19.2 (6.1)	18.4 (7.5)	0.7971
	2.a.2	2.4 (3.8)	3.8 (5.9)	0.5382
	2.a.3	1.4 (2.6)	0.8 (2.2)	0.5872
b. Margin	2.b.1	19.3 (6.4)	18.9 (7.8)	0.9013
	2.b.2	2.5 (3.7)	3.4 (6.1)	0.6974
	2.b.3	1.2 (2.9)	0.7 (2.2)	0.6698

3. Color match and translucency	3.1	12.8 (5.2)	12.6 (4.9)	0.9177
	3.2	7.4 (3.4)	8.1 (4.1)	0.6826
	3.3.1	1.3 (1.5)	2.0 (3.1)	0.5234
	3.3.2	0.4 (0.8)	0.2 (0.6)	0.5560
	3.3.3	0.4 (0.9)	0.5 (1.5)	0.8664
	3.3.4	0.7 (2.2)	0.9 (2.8)	0.8627

^*∗*^Scores not presented in the table received value zero.

**Table 4:** Sum from the opinion of the evaluators about which restoration was considered more esthetic.

	Evaluators																			
	1		2		3		4		5		6		7		8		9		10	
	SS	MS	SS	MS	SS	MS	SS	MS	SS	MS	SS	MS	SS	MS	SS	MS	SS	MS	SS	MS
Total teeth	12	11	12	11	12	11	13	10	13	10	13	10	10	13	9	14	10	13	10	13
Wilcoxon test (*p*-value)	0.855		0.709		0.855		0.584		0.584		0.584		0.584		0.361		0.584		0.584	

SS, single-shade composite; MS, multishade composite.

## Data Availability

Data can be made available upon request to the corresponding author.
